# Does Heat–Induced Activation of Mast Cells Contribute to Elevated Temperature–Induced Male Infertility?

**DOI:** 10.3390/pathophysiology33030049

**Published:** 2026-07-09

**Authors:** Ali Sadek, Yulia Khramtsova, Boris Yushkov

**Affiliations:** 1 Department of Biology and Fundamental Medicine, Ural Federal University Named After the First President of Russia B. N. Yeltsin, 620002 Ekaterinburg, Russia; sadek1996@mail.ru; 2 Cell Culture Laboratory, Federal Scientific Research Institute of Viral Infections “Virom”, 620030 Ekaterinburg, Russia; 3 Laboratory of Immunophysiology and Immunopharmacology, Institute of Immunology and Physiology of the Ural Branch of the Russian Academy of Sciences, 620049 Ekaterinburg, Russia; y.hramtsova@iip.uran.ru

**Keywords:** mast cells, microenvironment, stem cell niche, high temperature, testis, epididymis, seminal vesicles, animal model

## Abstract

Background: The microenvironment of spermatogonial stem cells plays a crucial role in determining their developmental trajectory. Mast cells (MCs) are an important component of this microenvironment and are present along the pathway of sperm production and maturation. Elevated temperature is associated with various disorders of male fertility and its effect on MCs has not been previously studied. Methods: The experiment was conducted on male Wistar rats, which were divided into four groups: intact, control, single, and repeated heat exposure. Morphofunctional and morphometric parameters of MCs were investigated in testes, epididymides, and seminal vesicles. Correlation analysis was performed between MC parameters, ejaculated and epididymal spermatozoa, and testosterone levels. Results: MCs become activated even after a single heat exposure, manifested by increased degranulation. Repeated exposures lead to an increase in MC numbers in the reproductive organs and an enhancement of their functional activity. Testicular MCs exhibit the highest sensitivity to elevated temperature, followed by epididymal MCs. These findings reveal organ-specific differences in MCs responses, which may be explained by the anatomical location of these organs. Correlation analysis revealed a negative relationship between MC morphofunctional parameters and sperm functional characteristics in epididymis and ejaculate, as well as testosterone levels. Conclusions: Heat exposure activates MCs in male reproductive organs, and the changes in their morphofunctional parameters support a potential role in heat-related male reproductive impairment that requires further evidence.

## 1. Introduction

It has been suggested that disruptions in stem cell microenvironment underlie many diseases, including male infertility [1,2]. However, the mechanisms behind these disruptions remain poorly understood. According to current understanding, the testicular microenvironment consists of Sertoli cells, Leydig cells, peritubular myoid cells, and testicular immune cells, including mast cells (MCs) [3]. The latter are of particular interest because multiple cellular components of the testicular microenvironment, including spermatogonial stem cells (SSCs) and germ cells, express receptors that respond to MC-derived mediators. The expression of histamine receptors on germ cells [4], histamine receptors and tryptase receptors (PAR2) on SSCs [5], and PAR2 receptors on spermatozoa [6] suggests that MC mediators may directly influence germ cell function. Moreover, the preferential localization of MCs near blood vessels, even under physiological conditions, together with the preferential positioning of SSCs near blood vessels adjacent to their niche, supports the hypothesis of their interaction.

Several studies have reported a significant increase in the number of testicular MCs in patients diagnosed with male infertility, especially in areas surrounding the seminiferous tubules [5]. This includes various forms of azoospermia, both obstructive and non-obstructive, idiopathic types of male infertility [7,8,9,10,11,12,13,14,15], oligozoospermia [7], varicocele [10,16], orchitis [17], mixed atrophy of the seminiferous tubules [18,19] and testicular fibrosis [20]. The increased density of MCs is also accompanied by changes in their subtypes. In pathological conditions, the predominant subtype contains both tryptase and chymase, whereas under normal physiological conditions, the tryptase-only subtype predominates [10].

MCs have also been detected in the ejaculate of infertile patients. Several studies indicate that mediators released from testicular MCs can negatively affect sperm motility, potentially contributing to asthenozoospermia [16,21,22]. For example, tryptase reduces sperm motility by acting on PAR-2 receptors located on the acrosome and midpiece of human spermatozoa [6]. 

Thus, the increase in the number of MCs and changes in their subtypes in the testes in various forms of male infertility indicate their potential involvement in the development of the pathology [3,23].

Factors that directly affect SSCs and other germ cells have been identified as etiological causes of male infertility. These factors include exposure to ionizing radiation [24], chemotherapeutic agents [25], and other chemical toxicants [26], which primarily induce DNA damage in germ cells and lead to their apoptosis. At the same time, many factors may indirectly impair male fertility by altering signaling interactions among cellular components of the testicular microenvironment [2]. Their etiology is often unclear and can account for a significant proportion of idiopathic male infertility, which itself represents up to 20% of all cases [27].

Among such factors, elevated testicular temperature can be highlighted [28]. Its negative effects on male fertility are well documented in the literature. Consistently, different models of scrotal hyperthermia across species show similar detrimental effects on sperm parameters. Moreover, clinical studies dating back to 1980 have demonstrated that scrotal cooling can be an effective approach that improves semen quality and natural pregnancy rates in patients with scrotal hyperthermia [29].

Increased scrotal temperature exerts both direct effects on germ cells and indirect effects by disrupting the testicular microenvironment. While its direct effects on germ cells have been comprehensively investigated [30], its indirect effects remain insufficiently studied [31]. Moreover, the response of MCs in male reproductive organs to this factor has not yet been investigated [31]. 

Experimental models based on Wistar rats offer translational relevance for human male fertility and infertility research due to many similarities in reproductive anatomy, hormonal regulation and physiological responses [32]. These similarities also include the comparable structure of the testicular microenvironment, including the presence of MCs [33]. Furthermore, as in most mammals, in both species the testes must be maintained at 2–8 °C below core body temperature to support normal spermatogenesis. Due to these similarities Wistar rats were used in our study to investigate the effect of heat exposure on MCs of male reproductive organs.

Different approaches can be used to model elevated scrotal temperature. In our study, we used a commonly applied approach that aims to overwhelm the scrotum’s ability to regulate testicular temperature, using warm water baths and exposure to elevated ambient air temperatures in a specially equipped thermostatic chamber.

In light of the above, the aim of this study is to investigate the response of MCs in the reproductive organs of male rats to elevated temperature and to explore the relationship between their morphofunctional parameters and spermatogenesis.

## 2. Materials and Methods

### 2.1. Animals and Ethics Statement

The present study was designed to explore the response of MCs in different male reproductive organs to heat exposure; hence, five or six animals were used per group and sample size was confirmed based on the resource equation method [34,35]. The calculation method is presented in Appendix A.1. The study protocol and all experimental procedures were reviewed and approved by the Ethics Committee of the Institute of Immunology and Physiology of the Ural Branch of the Russian Academy of Sciences (IIF UB RAS) (No. 10-23, dated 9 October 2023), in accordance with Directive 2010/63/EU of the European Parliament and the European Council and in compliance with the ARRIVE guidelines.

Sexually mature male Wistar rats weighing 310–440 g and aged 4 months (*n* = 31) were precured from the vivarium of IIF UB RAS. During the experiment, the animals were housed under standard vivarium conditions with a 12 h light/dark cycle, without a special diet and with free access to drinking water. Animals were monitored daily for signs of distress, including weight loss, reduced mobility, abnormal posture, and changes in grooming or feeding behavior. No distress or adverse events were observed.

### 2.2. Inclusion and Exclusion Criteria

Only healthy animals were included. Moreover, semen characteristics were evaluated prior to the experiment to ensure that only animals with normal spermatogenesis were enrolled. No animals were excluded during the course of the experiment.

### 2.3. Experimental Design

The experimental unit was the individual male rat. Animals were randomly allocated to four groups by drawing identification numbers from a container: (1) a group of intact animals (INT), *n* = 6; (2) a single local heat exposure group (ST), *n* = 5; (3) a control group for repeated heat exposure (RT(C)), *n* = 10; (4) an experimental group for repeated heat exposure (RT(X)), *n* = 10. A random subgroup from each repeated heat exposure group (sRT(C), *n* = 5; sRT(X), *n* = 5) was designated for fertility assessment. Animals in these subgroups were not euthanized on day 48 of repeated heat exposure (the experiment endpoint) and, therefore, were not included in the morphofunctional assessment of MCs.

### 2.4. Heat Exposure Modeling

Single local heat exposure was modeled by heating the lower body of the rat in a water bath for 30 min. The water temperature was continuously monitored and maintained at 43 ± 1 °C [36,37].

Repeated heat exposure was performed according to a previously established protocol [38], by placing the animals in a specially equipped thermostat set to 43 ± 1 °C [36,39], with maintained access to water and air. Animals were exposed for 30 min daily over 48 days, corresponding to the duration of one full spermatogenic cycle in rats [40]. The control group underwent the same procedures, but with the thermostat turned off and maintained at room temperature, thereby controlling for potential confounding factors associated with the experimental procedure (e.g., time spent in the thermostat, limited space inside the thermostat chamber).

The two experimental models differ not only in the heat exposure regimen but also in the method of exposure. The single exposure was applied using a water bath, which provides efficient and localized heat transfer to the lower body. In contrast, repeated heat exposure was conducted in a thermostat chamber (whole body exposure) to avoid repeated anesthesia associated with water bath procedures and to allow daily exposure over 48 days.

At the end of the experiment, ejaculate samples were collected. The animals were then euthanized by Isoflurane (Laboratories Karizoo, S.A., Barcelona, Spain) overdose. Epididymal seminal fluid was collected from each animal, along with the reproductive organs: testes, epididymides, and seminal vesicles.

### 2.5. Fixation and Staining

One organ from each pair was fixed in 10% buffered formalin for 48 h, followed by washing in running tap water for 2 h. Subsequently, standard histological processing was performed using a Leica TP 1020 automated tissue processor (Leica Biosystems, Nußloch, Germany), including six changes of graded ethanol of increasing concentration, three changes of xylene, and three changes of molten paraffin. The contralateral organ was fixed in Carnoy’s solution for 30 min, followed by two changes of ethanol and subsequent standard histological processing. Fixed tissues were embedded in paraffin using a Leica EG 1160 embedding station (Leica Biosystems, Nußloch, Germany). Sections of 4 µm thickness were prepared from cooled paraffin blocks of the organs using HM 450 sliding microtome (Thermo Scientific, Allentown, PA, USA).

Sections of organs fixed in 10% formalin were stained with toluidine blue to assess MCs number and functional status, represented by their degranulation. The protocol was unified for all sections. This includes the following steps: paraffin sections were deparaffinized in three changes of xylene (2 min each), followed by three changes of 96% ethanol (2 min each). Sections were then rinsed in distilled water (two changes, 2 min each) and stained with toluidine blue for 40 min. After staining, sections were rinsed in distilled water (two changes, 1 min each), dehydrated through three changes of 96% ethanol (2 min each), cleared in three changes of xylene (2 min each), and mounted using a permanent mounting medium.

Sections of organs fixed in Carnoy’s solution were stained with Alcian blue–safranin to evaluate the degree of granule maturation. The protocol was unified for all histological sections. This includes the following steps: paraffin sections were deparaffinized in three changes of xylene (2 min each), followed by three changes of 96% ethanol (2 min each). Sections were then rinsed in distilled water (two changes, 2 min each) and stained with Alcian blue–safranin for 15 min. After staining, sections were rinsed in distilled water (two changes, 1 min each), dehydrated through three changes of 96% ethanol (2 min each), cleared in three changes of xylene (2 min each), and mounted using a permanent mounting medium. 

The staining process was performed using a Leica ST5010 automated histological staining system (Leica Biosystems, Nußloch, Germany).

All samples were coded by an independent laboratory technician to ensure that the investigator was blinded to group allocation throughout the study, including during statistical analysis.

### 2.6. Quantification of MC Density

Scans of prepared tissue sections were obtained using a Leica DM2500 light microscope (Leica Microsystems, Wetzlar, Germany) equipped with a Basler acA1920-40um camera (Basler, Ahrensburg, Germany) and MultiMedia Catalog 2008–2020 software. Sections were imaged under standardized scanner and camera settings using a 20× objective with a pixel size of 0.293 × 0.293 μm (width × height). The resulting images were analyzed using QuPath 0.5.0 [41], which can read metadata provided by the MultiMedia Catalog software; therefore, no additional calibration was required. MCs were quantified across the entire tissue section using the brush tool to define the analyzed area and the point tool to mark individual MCs within this area in QuPath. MC density was then calculated as the number of MCs per 1 mm^2^ of tissue section.

### 2.7. Assessment of MC Synthetic Activity and Morphometric Parameters

FIJI ImageJ 1.54f [42] was used to objectively assess MC synthetic activity and morphometric parameters. All scans were first opened in QuPath, then imported into ImageJ via the QuPath ImageJ extension and saved as TIFF files. The saved TIFF images preserved the metadata of the original scans because QuPath provides this information to ImageJ; therefore, no additional calibration (pixel-to-micron conversion) was required. To ensure the accuracy of software metadata-based calibration, this calibration was independently verified using a stage micrometer with a known scale. The micrometer was imaged under the same standardized conditions as the experimental samples. First, the resulting image was opened in QuPath, then exported to ImageJ via the QuPath ImageJ extension and saved as a TIFF file. The TIFF image was subsequently opened in FIJI ImageJ. In both software environments, the distance between defined scale divisions on the micrometer was measured, and the obtained values were compared with the known physical distance on the stage micrometer.

**Figure A1:**
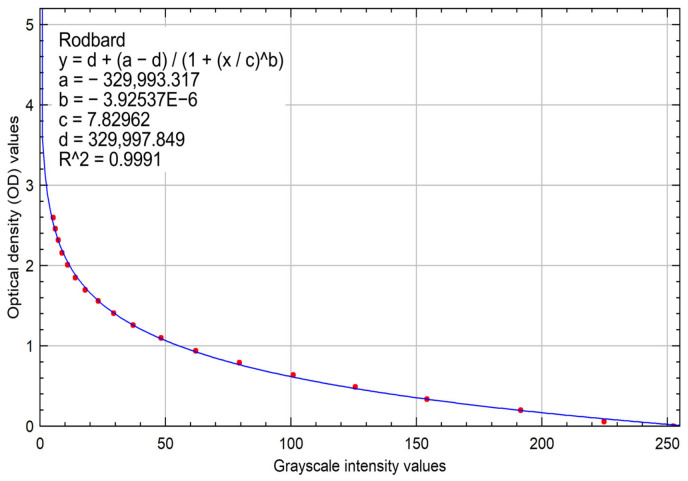
ImageJ calibration curve used for measuring mast cells’ optical density (OD_MC_).

Before analysis, FIJI ImageJ was calibrated using the Rodbard function (Figure A1) to convert grayscale intensity values to optical density (OD) values. All TIFF images were then converted to 8-bit grayscale. In each scan, the OD of 50 cells was measured; if fewer than 50 cells were present, all available cells were measured. To account for potential confounding factors such as section thickness, fixation time, and staining duration, all samples were processed using a standardized fixation and staining protocol as described previously. In addition, the staining intensity of MCs was normalized to the staining intensity of adjacent areas of connective tissue (CT) in 20 random fields on each section. The ratio of MC optical density (OD_MC_) to CT optical density (OD_CT_) was then calculated, indicating the synthetic activity of the cells. A higher OD_MC_/OD_CT_ ratio reflects greater synthetic activity of MCs. However, because the properties of CT may vary between different organs, normalization was not performed when comparing synthetic activity across organs—only the OD_MC_ values were used. Finally, cell-level measurements of OD_MC_, OD_MC_/OD_CT_ ratio, area, and perimeter were averaged to obtain animal-level values for statistical analysis.

### 2.8. Assessment of MC Functional Activity

**Figure 1:**
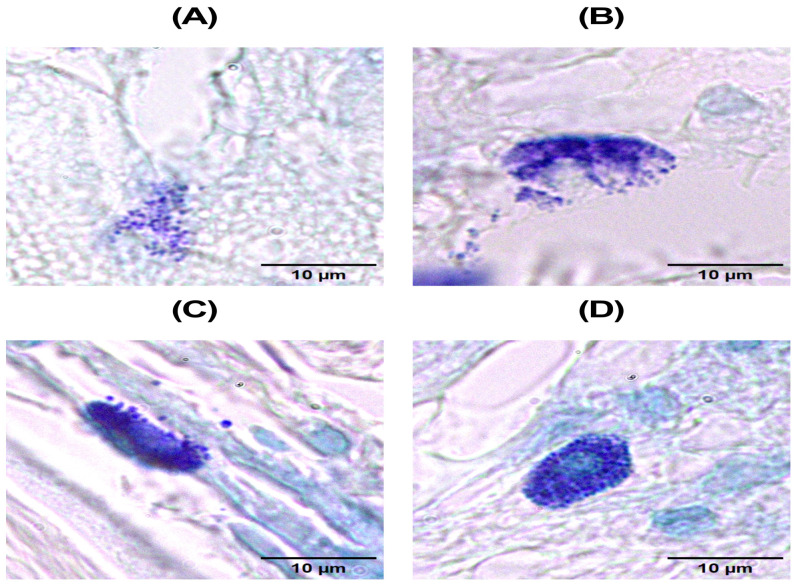
Different degrees of mast cell degranulation in the rat testis. (**A**) strong; (**B**) moderate; (**C**) weak; (**D**) absent. Staining with Toluidine blue. Magnification ×100, oil immersion.

MCs were classified into four categories (Figure 1): inactive, weakly, moderately, and actively degranulating. The assessment was performed using a 100× oil immersion objective to ensure accurate classification.

The degranulation index (DI) of MCs was calculated using the following formula:

DI = D/(D + I) × 100,(1)

where D represents the number of MCs with clear signs of degranulation and I represents the number of inactive MCs.

### 2.9. Assessment of MC Granule Maturity

**Figure 2:**
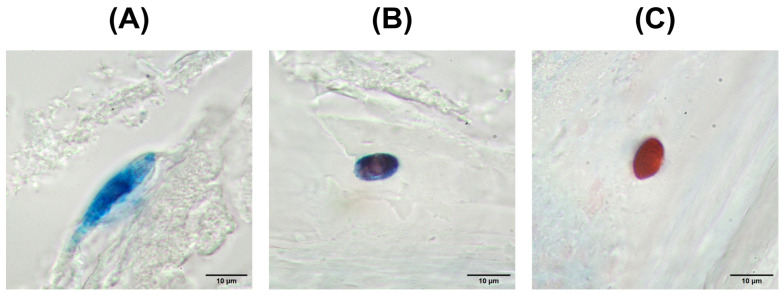
Mast cells in the rat testis with different degrees of granule maturity. (**A**) with immature granules; (**B**) with intermediate granules; (**C**) with mature granules. Staining with Alcian blue—safranin. Magnification ×100, oil immersion.

Using Alcian blue—safranin staining, MCs were classified into three groups (Figure 2): (1) MCs with immature granules, stained blue due to the affinity of their components for Alcian blue alone (Alc^+^ granules); (2) MCs with granules of intermediate maturity, stained purple due to the affinity of their components for both Alcian blue and safranin (Alc^+^ Saf^+^ granules); (3) MCs with mature granules, stained red due to the high content of heparin, a sulfated glycosaminoglycan with a strong affinity for safranin (Saf^+^ granules). After classification, the percentage of each group was calculated.

### 2.10. Ejaculated Sperm Collection

Ejaculate was collected using oxytocin-induced ejaculation method as previously described [38,43]. Rats were lightly sedated with brief exposure to isoflurane to facilitate ejaculate collection. Then 0.2 mL of oxytocin solution (5 IU/mL; Moscow Endocrine Plant, Moscow, Russia) was administered intraperitoneally using an insulin syringe. The prepuce was gently retracted and manually stimulated by repeated hand movements and held in position until ejaculation occurred. The ejaculate was collected into a 1.5 mL Eppendorf tube (MiniMed, Bryansk, Russia) using a micropipette, and its volume was measured. The sample was then diluted 20-fold with 0.9% sodium chloride solution at room temperature. Semen analysis was subsequently performed.

### 2.11. Epididymal Sperm Collection

Following euthanasia by isoflurane overdose, the epididymides were surgically excised. To obtain an epididymal sperm suspension, equal-sized segments of the cauda epididymides were cut. The tissue was transferred into a glass test tube containing 2 mL of 0.9% sodium chloride solution prewarmed to 37 °C. The tube was vigorously shaken and incubated at 37 °C for 10 min. After incubation, the suspension was shaken again to ensure homogeneity then a sample was taken for sperm analysis.

### 2.12. Evaluation of Sperm Functional Parameters

Sperm concentration was assessed by counting spermatozoa in five large squares of hemocytometer (MiniMed, Bryansk, Russia) positioned diagonally across the counting chamber. The count was conducted in two chambers, then the mean value was calculated. Sperm concentration was calculated using the following formula:

Sperm concentration (10^6^/mL) = A × df × 1000/B × 0.004, (2)

where A—the number of spermatozoa counted, df—the dilution factor, and B—the number of squares counted.

Sperm motility was determined by counting the number of motile and total spermatozoa in five randomly selected microscopic fields. The percentage of motile spermatozoa was calculated as follows:

Motility (%) = B/C × 100,(3)

where B is the number of motile spermatozoa and C is the total number of spermatozoa counted.

Sperm agglutination was assessed throughout the entire counting chamber using the following scoring system: 0 point—no agglutination was observed; 1 point—up to 10–15% of spermatozoa aggregated into small clumps; 2 points—up to 50% of spermatozoa aggregated into both small and large clumps; 3 points—extensive agglutination, with nearly all spermatozoa forming large aggregates.

The abundance of lecithin granules was evaluated throughout the counting chamber using a semi-quantitative scoring system: 0 point—no lecithin granules observed; 1 point—low abundance; 2 points—moderate abundance; 3 points—high abundance.

### 2.13. Serum Testosterone Measurement

Serum testosterone concentration was determined using an automated enzyme-linked immunosorbent assay (ELISA) analyzer (Lazurite, Dynex Technologies, Chantilly, VA, USA) and a commercial ELISA kit for the quantitative determination of total serum testosterone (Testosterone-IMAXIZ, Vital Development, St. Petersburg, Russia), according to the manufacturer’s instructions.

### 2.14. Fertility Assessment

The two previously mentioned subgroups (sRT(C), *n* = 5 and sRT(X), *n* = 5) were used to explore the fertilizing ability of male rats after repeated heat exposure. For this purpose, each male was housed with two 3-month-old females. The presence of pregnancy, the duration of gestation, and the number of pups born were recorded.

### 2.15. Statistical Analysis

All data were processed using GraphPad Prism 9.5.1. The data of all groups were tested for normality of distribution using the Shapiro–Wilk test. Due to the methodological differences between the two heat exposure models (single vs. repeated heat exposure), statistical analyses were performed separately for each model. The Mann–Whitney U test was used for pairwise comparisons, particularly between INT and ST groups and between sRT(C) and sRT(X) for fertility assessment.

For comparisons among the INT, RT(C), and RT(X) groups, the Kruskal–Wallis test was performed, followed by Dunn’s multiple comparisons test. To control for multiple comparisons, *p*-values were adjusted using the two-stage step-up procedure of Benjamini, Krieger, and Yekutieli, yielding false discovery rate (FDR)-adjusted *p*-values (*q*-values), as recommended in GraphPad Prism. The primary comparisons of interest were INT vs. RT(C) and between RT(C) vs. RT(X). However, all pairwise comparisons among the three groups are presented.

Differences were considered statistically significant at *p* < 0.05 (or *q* < 0.05 when correction was applied). Data are presented as median with interquartile range [Q1; Q3].

The rank-biserial correlation coefficient was used to estimate the effect size for differences identified by the Mann–Whitney U test. The following formula was applied:

*r_rb_* = δ = 1 − (2 × U/n1 × n2), (4)

where *r_rb_*—Rank-biserial correlation coefficient, δ—Cliff’s delta, U—the Mann–Whitney U statistic, and where n1 and n2 are the respective sample sizes of the two independent groups.

The effect size (*r*) was calculated for differences identified by Dunn’s post-hoc test using the following formula:

*r* = Z/√N, (5)

where Z represents the standardized test statistic from Dunn’s test and N represents the total number of observations in the two groups being compared.

The effect size values were interpreted as follows: around 0.1 indicates a small effect, 0.3 a medium effect, and 0.5 or higher a large effect [44].

Pairwise comparisons are provided in Table A1 and Table A2.

Correlation analysis was conducted using Spearman’s correlation coefficient to explore the relationships between MC parameters (number and degranulation) and sperm parameters (concentration, motility, agglutination), as well as blood testosterone levels. All experimental groups were included in this analysis. The correlation *p*-values were corrected using the two-stage stepwise method of Benjamini, Krieger, and Yekutieli as recommended by the statistical software. Correlation strength was interpreted based on Spearman correlation coefficient magnitude (weak: |*r*| < 0.3; moderate: 0.3 ≤ |*r*| < 0.5; strong: |*r*| ≥ 0.5). Statistical information of correlation analyses is presented in Table A3.

## 3. Results

### 3.1. Morphofunctional Parameters of MCs in Different Reproductive Organs of Male Rats

In intact animals, MCs were identified in all examined reproductive organs. In the testes, they were located in the connective tissue of the tunica albuginea, whereas in the epididymides and seminal vesicles they were distributed within the connective tissue and stroma, often near blood vessels. The morphofunctional parameters of MCs varied depending on the organ in which they were located. Specifically, the testes contained the lowest number of MCs, which were also the smallest in size and exhibited the least synthetic activity when compared with MCs of other reproductive organs. In contrast, the seminal vesicles contained the highest number of MCs, with the greatest synthetic activity and largest cell size. Under normal conditions, no differences were observed in the proportions of MCs with different degrees of granule maturity (Table 1).

### 3.2. Effects of Single Heat Exposure on MC Parameters

**Figure 3:**
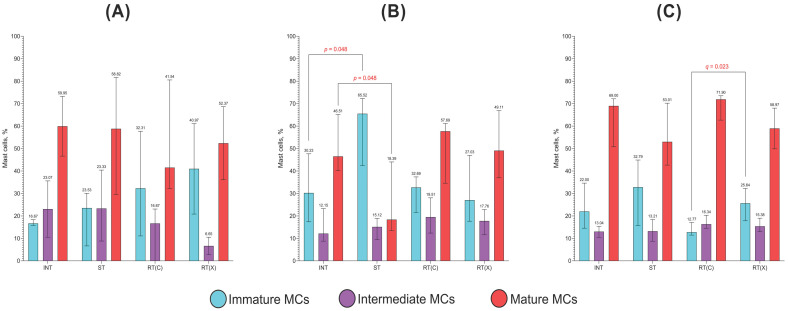
Mast cells with different degrees of granule maturity in the reproductive organs of male rats. (**A**) Testis; (**B**) epididymis; (**C**) seminal vesicle. Data are presented as median with interquartile range [Q1; Q3]. Testis: *n* = 5 per group; epididymis: *n* ≥ 5 per group; seminal vesicle: *n* = 5 per group. Significant differences between INT and ST groups were determined through the Mann–Whitney U test (*p* < 0.05). Significant differences between INT, RT(C) and RT(X) groups were determined through Dunn’s test and were adjusted using the two-stage step-up method of Benjamini, Krieger, and Yekutieli (*q* < 0.05). All comparisons are shown in Table A2. INT—intact group; ST—single heat exposure group; RT(C)—repeated heat exposure control group; RT(X)—repeated heat exposure experimental group; MCs—mast cells.

After a single exposure to elevated temperature, testicular MCs showed an increase in degranulation compared with the intact group (Table 2). In the epididymides, the proportion of MCs with different granule maturity levels changed, with an increase in the number of MCs containing immature granules (Figure 3B). In the seminal vesicles, an increase in MC degranulation was observed (Table 2).

### 3.3. Effects of Repeated Heat Exposure on MC Parameters

Repeated heat exposure resulted in pronounced alterations in MCs’ morphofunctional characteristics across all reproductive organs. The number of testicular MCs increased in comparison with the control group (Table 3). In contrast, in the control group, they showed a decrease in density compared with the intact animals. No differences in the proportions of MCs with different granule maturity levels were found between the groups (Figure 3A).

In the epididymides, MC response was similar to that observed in the testes, characterized by an increased number (Table 3). Consistently, no differences in the proportions of MCs with different granule maturity levels were detected between groups (Figure 3B).

MCs in the seminal vesicles responded to elevated temperature differently from those in the testes and epididymides. While no statistically significant change in their number was observed, their synthetic activity and area decreased compared with the control group (Table 3). Moreover, the number of cells with immature granules increased (Figure 3C). In contrast, the control group displayed an increase in MC size relative to intact animals (Table 3).

**Figure 4:**
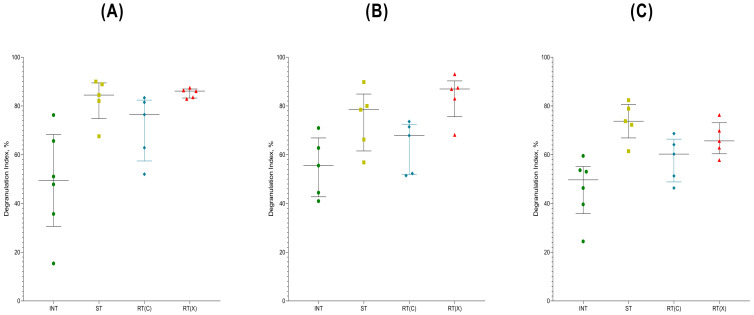
Mast cell degranulation in reproductive organs after repeated heat exposure. Data are presented as median with interquartile range [Q1; Q3]. (**A**) Testis; (**B**) epididymis; (**C**) seminal vesicles. *N* ≥ 5 per group. INT—intact group; ST—single heat exposure group; RT(C)—repeated heat exposure control group; RT(X)—repeated heat exposure experimental group.

Although no statistically significant differences in the degranulation of testicular and epididymal MCs were observed after repeated heat exposure, both groups showed a tendency toward increased degranulation, with large effect sizes of 0.57 and 0.60, respectively (Figure 4A,B), where in most animals are located at higher degranulation values and show reduced variability in their responses. However, the uncertainty around the estimates precludes firm conclusions.

### 3.4. Effects of Repeated Heat Exposure on Male Fertility Outcomes

**Figure 5:**
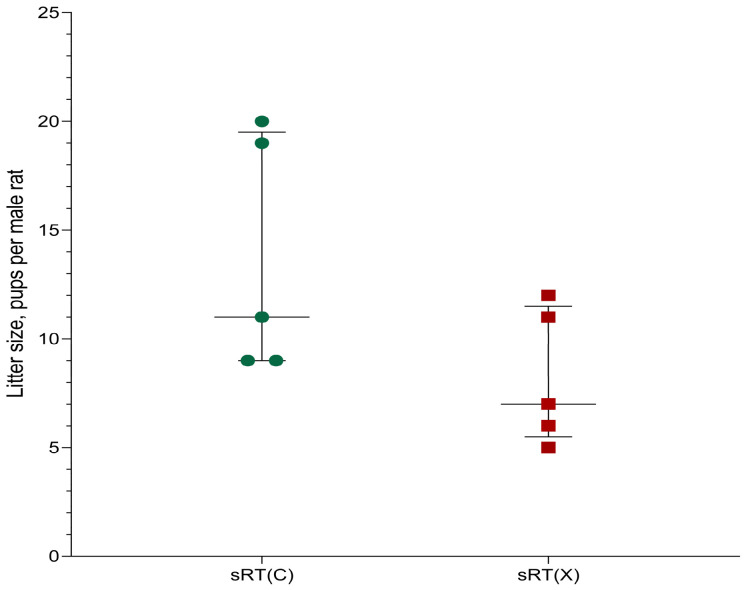
Assessment of fertility in male rats following repeated heat exposure. Data are presented as median with interquartile range [Q1; Q3]. *N* = 5 per group. No significant difference was observed using the Mann–Whitney U test, *p* = 0.1825. The effect size was calculated using the rank-biserial correlation coefficient, *r_rb_* = 0.56. sRT(C)—repeated heat exposure control subgroup; sRT(X)—repeated heat exposure experimental subgroup.

After co-housing males with females, pregnancy was confirmed in both the control and heat-exposed experimental groups. No significant differences in gestation duration and litter size were observed (Figure 5). 

### 3.5. Correlation Analysis Between MC Parameters and Spermatogenesis Indicators

MCs are present along the sperm production and maturation pathway. To explore the association between MC morphofunctional changes and spermatogenic disturbances under heat stress, correlation analyses were performed between MC parameters in a given organ and the sperm located downstream in the maturation pathway, i.e., after potential MC-mediated effects.

**Figure 6:**
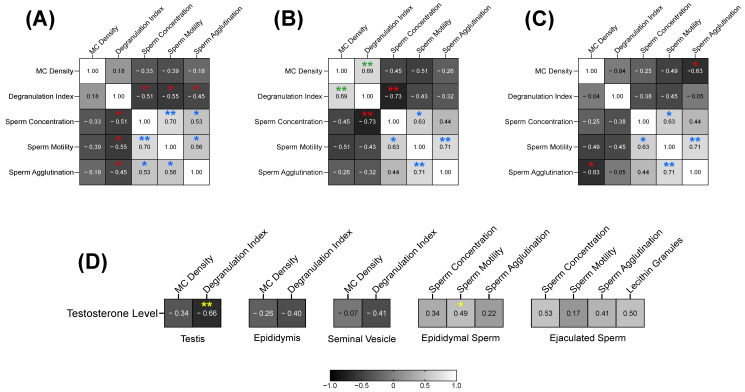
Correlation between mast cell parameters, sperm parameters, and testosterone level. (**A**) Testicular mast cells and epididymal sperm (*n* = 20); (**B**) epididymal mast cells and ejaculated sperm (*n* = 15); (**C**) seminal vesicle mast cells and ejaculated sperm (*n* = 15); (**D**) testosterone with mast cell parameters in all reproductive organs (*n* = 20) and sperm parameters (epididymal sperm, *n* = 20; ejaculated sperm, *n* = 15). Data are presented as correlation matrices showing Spearman correlation coefficients (*r*). Statistical significance was defined as *q* < 0.05 (*p*-values were adjusted using the two-stage stepwise method of Benjamini, Krieger, and Yekutieli): *—*q* < 0.05; **—*q* < 0.01. Green asterisk—statistically significant correlation between two different mast cell parameters; red asterisk—statistically significant correlation between a mast cell parameter and a sperm parameter; blue asterisk—statistically significant correlation between two different sperm parameters; yellow asterisk—statistically significant correlation between testosterone level and mast cell parameters in reproductive organs and sperm parameters in epididymis and ejaculate. Correlation strength was interpreted as follows: weak |*r*| < 0.3; moderate: 0.3 ≤ |*r*| < 0.5; strong: |*r*| ≥ 0.5). Correlation statistical results are presented in Table A3. MC—mast cells. Note: Correlations were calculated using pooled data from all experimental groups and may therefore partly reflect the overall effects of heat exposure in addition to associations between the analyzed variables.

Correlation analysis between testicular MC parameters and sperm parameters in the epididymides revealed a negative correlation between testicular MC degranulation and all sperm parameters (Figure 6A). Similarly, correlation analysis between epididymal MC parameters and ejaculated sperm parameters showed a strong negative correlation between MC degranulation and sperm concentration (Figure 6B). Finally, correlation analysis between seminal vesicle MC parameters and ejaculated sperm parameters demonstrated a negative correlation between the number of MCs in the seminal vesicles and sperm agglutination (Figure 6C).

Correlation analysis between testosterone levels and MC parameters in the reproductive organs—considering that the testes are androgen-producing organs, whereas the epididymides and seminal vesicles are androgen-dependent [45,46,47]—revealed a negative relationship between testicular MC degranulation and testosterone levels. In contrast, MCs in the epididymides and seminal vesicles did not show such a relationship (Figure 6D). Analysis of testosterone levels and sperm parameters in the epididymides and ejaculate demonstrated a positive correlation between hormone concentration and sperm motility in the epididymides (Figure 6D).

## 4. Discussion

Under physiological conditions, MCs were observed in all male reproductive organs, along the entire pathway of sperm production, maturation, and storage—from the testicular microenvironment surrounding the SSC niche, through the epididymis where sperm mature and are stored, and in accessory reproductive glands (seminal vesicles). However, they differ in number, synthetic activity and size.

In the testes, MCs are the fewest in number, the smallest in size, and have the weakest synthetic capacity in comparison with MCs of other reproductive organs. This may be necessary to maintain the immune-privileged status of the testis, which is a critical requirement for spermatogenesis.

In the epididymis, MCs are more abundant than in testis and are more synthetic and larger in size. Leung et al. [48] have suggested that epididymal MCs regulate electrolyte and fluid secretion in the epididymis through the release of 5-hydroxytryptamine (serotonin). In addition, they may also influence the mechanical function of the epididymis by secreting histamine, which induces phasic contractions of the distal cauda epididymis via histamine receptors 2 (H_2_R) and autonomic neurotransmitters [49]. 

In the seminal vesicles, MCs are the most abundant among all studied reproductive organs; they are large and highly active in synthesis. This differences may be related to the distinct anatomical locations of the reproductive organs: the seminal vesicles are located inside the body, whereas the testes and their epididymides are situated outside the abdominal cavity.

Moreover, the higher number and synthetically active state of MCs in the epididymis and seminal vesicles under normal conditions, when compared with the testes, suggest that MCs may carry out different or additional regulatory functions in these two organs than they do in the testes.

Testicular MCs also change with age, as indicated in our study by the observed decrease of their number in control groups when compared with the intact group (the control animals were 48 days older than the intact ones due to the experimental modeling of repeated heat exposure). The age-related changes have also been reported previously in the literature [33].

Elevated temperature is a negative factor for spermatogenesis and can adversely affect not only the germ cells directly, but also the components of the testicular microenvironment [31], including MCs.

A single local exposure to elevated temperature activates MCs, specifically in testes and seminal vesicles, as indicated by an increase in their degranulation index. At the same time, the differences in MC parameters among these organs after a single exposure remain the same as those under physiological conditions. This suggests that MCs in all examined organs exhibit a similar response to heat exposure. 

However, an increased proportion of epididymal MCs with immature granules was observed. As no increase in epididymal MC density was detected after single exposure, this increase may be attributed to the rapid, tissue-specific (only observed in the epididymis) beginning of a regranulation process following active degranulation [50] in response to heat exposure. It is worth noting that no statistically significant difference in epididymal MC degranulation was detected between the intact and single heat exposure groups. Therefore, this interpretation remains speculative and requires further investigation. 

Repeated exposure to elevated temperature led to a significant increase in the number of testicular and epididymal MCs. This indicates the migration of MCs into these organs. The most pronounced increase was observed in the testes, where MCs increased threefold, while in the epididymis they nearly doubled.

Moreover, repeated heat exposure induced activation of testicular and epididymal MCs, as indicated by a tendency toward increased degranulation (Figure 4). Although our findings are descriptive, they are consistent with previous in vitro studies investigating the mechanisms underlying heat-induced MC activation. Those studies demonstrated that a temperature of 43 °C directly activates HMC-1 cells, resulting in their degranulation, an increase in intracellular calcium concentration [Ca^2+^]_i_, and ATP secretion. The molecular mechanism underlying this response involves activation of transient receptor potential vanilloid 1 (TRPV1), a thermosensitive channel protein expressed on MCs, which has a thermal threshold of 43 °C [51]. Moreover, higher temperatures (above 50 °C) have also been reported to induce MC degranulation, with TRPV2 additionally contributing to their activation [52,53].

The lack of change in the proportions of testicular and epididymal MCs with granules at different stages of maturity after repeated exposure, despite the observed increase in MC number due to migration and their continuous degranulation, may suggest the existence of mechanisms that maintain these proportions.

Thus, testicular MCs are highly sensitive to elevated temperatures, followed by epididymal MCs, which show a nearly identical response. In contrast, MCs in the seminal vesicles exhibit the lowest level of response after repeated exposure. This indicates organ-specific differences in MC responsiveness, likely explained by the anatomical locations of these organs, inside versus outside the body, as seminal vesicles function at core body temperature, in contrast to the testes and epididymides.

As reported in our previous work, repeated exposure to elevated temperature leads to a significant impairment of spermatogenesis [54], which is manifested by a decrease in ejaculated sperm concentration (RT(C): 74.60 × 10^6^ ± 9.98 vs. RT(X): 11.40 × 10^6^ ± 5.13, *p* = 0.001; *r* = 0.92), sperm motility (RT(C): 43.0% ± 4.23 vs. RT(X): 7.10% ± 4.01, *p* < 0.001; *r* = 0.85), agglutination (RT(C): 2.20 ± 0.20 vs. RT(X): 0.50 ± 0.27, *p* = 0.005; *r* = 0.83), and lecithin granules (RT(C): 2.30 ± 0.15 vs. RT(X): 0.60 ± 0.22, *p* = 0.002; *r* = 1.02), as well as by a decrease in epididymal sperm concentration (RT(C): 143 × 10^6^ ± 18.07 vs. RT(X): 42 × 10^6^ ± 6.04, *p* = 0.004; *r* = 0.83), sperm motility (RT(C): 57.40% ± 6.27 vs. RT(X): 17% ± 2.97, *p* = 0.028; *r* = 0.85), and agglutination (RT(C): 2.00 ± 0.00 vs. RT(X): 0.40 ± 0.24, *p* = 0.017; *r* = 0.71), as well as a reduction in serum testosterone levels (RT(C): 109.91 ± 7.42 vs. RT(X): 42.79 ± 9.23, *p* = 0.010; *r* = 0.78).

Despite disturbances in spermatogenesis parameters after repeated heat exposure, fertility in rats was not significantly affected (*p* = 0.183). Given the limited number of rats used for this assessment, a confirmatory conclusion cannot be made and the fertility evaluation remains exploratory. Moreover, fertility assessments in laboratory animals have limited sensitivity. According to the Guidelines for Reproductive Toxicity Risk Assessment (United States Environmental Protection Agency, USA) [55], sperm production can be reduced by up to 90% without detectable impairment of fertility in some rat and mouse strains, including Wistar rats. Conversely, smaller reductions in sperm quality or quantity can cause infertility in humans.

The correlation analysis showed negative relationships between the morphofunctional parameters of the MCs present in the reproductive organs and all epididymal and ejaculated sperm parameters, including sperm concentration, sperm motility and sperm agglutination. Moreover, a negative association between testicular MC degranulation and testosterone levels was observed. Furthermore, the negative indices may also reflect the overall effect of heat exposure rather than an independent relationship between MC parameters and sperm or testosterone outcomes.

Heat stress is known to directly affect Leydig cells, causing morphological alterations and impairing testosterone production [31]. In addition, heat-induced activation of MCs may contribute to impaired testosterone synthesis through reciprocal interactions with Leydig cells mediated by histamine and androgen receptors [56,57,58,59,60,61].

The relationship between plasma testosterone levels and sperm parameters is manifested in our study as a moderate positive correlation with sperm motility in the epididymis. Previous studies have not identified a consistent association between testosterone levels and semen parameters [62,63]. However, several studies have reported a positive correlation between testosterone levels and motility [64,65,66], morphology [67,68] or both parameters [69]. 

## 5. Conclusions

Spermatogenesis is not governed by a single factor but by a complex network of interactions among the cellular components of the testicular microenvironment. Furthermore, the successful outcome of this process, reflected by the production of functional spermatozoa capable of fertilizing an oocyte, depends not only on events within the testes but also on subsequent sperm maturation in the epididymis and the provision of supportive secretions from the accessory sex glands. Consequently, disruption at any stage of this reproductive sequence can impair sperm quality and fertility. This is particularly relevant for MCs, which synthesize, store, and release a wide range of bioactive mediators upon activation, thereby potentially influencing multiple components of the male reproductive system.

**Figure 7:**
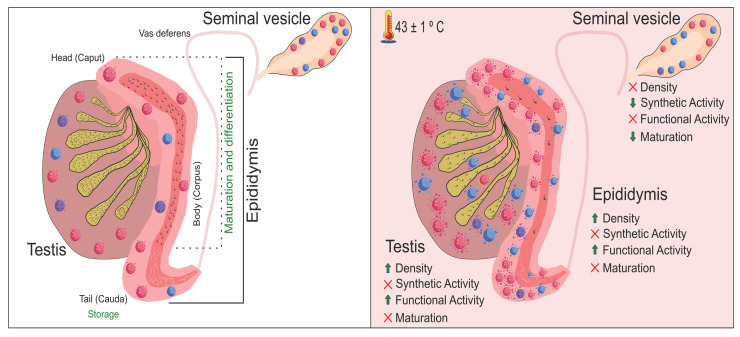
The effect of repeated heat exposure on mast cells distributed along the sperm formation and maturation pathway.

In this study, the migration and activation of MCs in male reproductive organs in response to heat exposure were demonstrated for the first time (Figure 7). Our findings suggest that MCs may contribute to heat-induced impairment of spermatogenesis, including forms of male infertility associated with elevated scrotal temperature, such as varicocele and cryptorchidism. However, the mechanisms underlying MC response and its consequences on spermatogenesis microenvironment were not investigated in the present study and need further research. Although the total number of animals in the present study exceeded the upper limit recommended by the resource equation method for the overall experimental design, several analyses, particularly the fertility assessment and single heat exposure model, were based on relatively small numbers of animals (*n* = 5 per group). Consequently, these findings require confirmation in larger studies.

Differences between the repeated heat exposure control group and the intact group were observed. We suggest that these differences may reflect age-related effects, therefore, future studies should include a separate age-matched control group.

Negative associations between MCs in male reproductive organs, sperm functional characteristics and testosterone levels were revealed. However, it is important to note that these correlation analyses should be considered exploratory, given the limited number of animals used combined with the inclusion of all experimental groups in the correlation analysis, the complex interplay of numerous cellular components involved in spermatogenesis, and the direct detrimental effects of heat exposure on sperm function.

## Figures and Tables

**Table 1:** Morphofunctional parameters of mast cells in the reproductive organs of intact male rats.

Parameters	Testis	Epididymis	Seminal Vesicle
MC density [cells/mm^2^]	0.30 (0.25–0.40)	2.04 (1.85–3.24)	7.90 (4.79–14.22) ^a^
Synthetic activity [OD_MC_/OD_CT_]	0.31 (0.30–0.34)	0.38 (0.37–0.41) ^a^	0.46 (0.39–0.57) ^a^
Degranulation index [%]	49.45 (30.63–68.29)	55.56 (42.71–66.88)	49.69 (35.83–55.13)
Area [μm^2^]	36.27 (31.35–45.79)	63.90 (55.32–72.84) ^a^	74.78 (60.70–84.68) ^a^
Perimeter [μm]	25.36 (24.33–26.73)	31.87 (29.69–33.66) ^a^	33.57 (30.44–36.23) ^a^
**Maturation degree of MC granules** **[%]**			
	**Testis**	**Epididymis**	**Seminal Vesicle**
Immature	16.67 (15.79–18.34)	30.23 (17.39–47.66)	22.00 (14.55–34.63)
Intermediate	23.07 (10.53–35.61)	12.15 (8.77–23.26)	13.04 (10.49–15.39)
Mature	59.95 (46.65–73.25)	46.51 (40.19–65.22)	69.00 (50.79–72.14)

Data are expressed as median with interquartile range [Q1; Q3]. *N* ≥ 5 per group. Significant differences between groups were determined through Dunn’s test and were adjusted using the two-stage step-up method of Benjamini, Krieger, and Yekutieli (*q* < 0.05): ^a^—indicates a significant difference in comparison to the testis. *q*-values and effect sizes are shown in Table A1. MC—mast cells; OD_MC_—mast cell optical density; OD_CT_—connective tissue optical density.

**Table 2:** Morphofunctional parameters of mast cells after single heat exposure.

	Testis		Epididymis		Seminal Vesicle	
	INT	ST	INT	ST	INT	ST
MC density [cells/mm^2^]	0.30 (0.25–0.40)	0.24 (0.23–0.42)	2.04 (1.85–3.24)	2.50 (0.66–3.43)	7.90 (4.79–14.22)	3.97 (3.44–5.83)
Synthetic activity [OD_MC_/OD_CT_]	3.36 (3.11–3.72)	3.16 (2.44–3.47)	4.86 (3.86–5.11)	4.89 (4.29–5.10)	5.71 (5.20–5.91)	4.99 (4.72–5.53)
Degranulation index [%]	49.45 (30.63–68.29)	84.48 (74.81–89.45) ^a^	55.56 (42.71–66.88)	78.46 (61.55–84.90)	49.69 (35.83–55.13)	73.75 (66.88–80.62) ^a^
Area [μm^2^]	36.27 (31.35–45.79)	49.26 (43.00–53.90)	63.90 (55.32–72.84)	71.06 (60.10–73.90)	74.78 (60.70–84.68)	73.98 (70.41–79.38)
Perimeter [μm]	25.36 (24.33–26.73)	28.59 (25.91–29.37)	31.87 (29.69–33.66)	31.64 (30.89–33.10)	33.57 (30.44–36.23)	35.08 (33.33–35.86)

Data are presented as median with interquartile range [Q1; Q3]. *N* ≥ 5 per group. Significant differences between groups were determined through the Mann–Whitney U test: ^a^—indicates a significant difference in comparison to INT group (*p* < 0.05). *p*-values and effect sizes are shown in Table A2. INT—intact group; ST—single heat exposure group; MC—mast cells; OD_MC_—mast cell optical density; OD_CT_—connective tissue optical density.

**Table 3:** Morphofunctional parameters of mast cells after repeated heat exposure.

		MC Density [cells/mm^2^]	Synthetic Activity [OD_MC_/OD_CT_]	Degranulation Index [%]	Area [μm^2^]	Perimeter [μm]
Testis	INT	0.30 (0.25–0.40)	3.36 (3.11–3.72)	49.45 (30.63–68.29)	36.27 (31.35–45.79)	25.36 (24.33–26.73)
	RT(C)	0.17 (0.09–0.22) ^a^	3.72 (3.01–4.24)	76.47 (57.43–82.41)	49.13 (40.92–52.83)	27.96 (25.07–29.86)
	RT(X)	0.39 (0.25–0.71) ^b^	3.71 (3.48–4.55)	86.11 (83.27–86.96) ^a^	39.57 (34.69–57.09)	26.48 (24.58–32.28)
Epididymis	INT	2.04 (1.85–3.24)	4.86 (3.86–5.11)	55.56 (42.71–66.88)	63.90 (55.32–72.84)	31.87 (29.69–33.66)
	RT(C)	2.92 (1.51–3.50)	4.57 (4.14–5.27)	67.86 (51.87–72.52)	74.60 (64.38–80.01)	33.89 (31.54–34.81)
	RT(X)	4.72 (4.27–6.69) ^a,b^	5.27 (4.84–5.36)	86.96 (75.60–90.28) ^a^	72.46 (67.87–91.72)	32.61 (32.22–37.84)
Seminal vesicle	INT	7.90 (4.79–14.22)	5.71 (5.20–5.91)	49.69 (35.83–55.13)	74.78 (60.70–84.68)	33.57 (30.44–36.23)
	RT(C)	6.90 (3.92–9.78)	5.58 (5.11–5.72)	60.28 (48.83–66.41)	90.42 (87.79–94.82) ^a^	36.78 (35.99–37.01)
	RT(X)	11.50 (7.11–21.73)	3.69 (3.27–3.90) ^a,b^	65.66 (60.38–73.11) ^a^	81.10 (76.67–83.43) ^b^	35.73 (34.69–36.26)

Data are presented as median with interquartile range [Q1; Q3]. *N* ≥ 5 per group. Significant differences between groups were determined through Dunn’s test and were adjusted using the two-stage step-up method of Benjamini, Krieger, and Yekutieli (*q* < 0.05): ^a^—indicates a significant difference in comparison to the intact group; ^b^—indicates a significant difference in comparison to the control group. *q*-values and effect sizes are shown in Table A2. MC—mast cells; OD_MC_—mast cell optical density; OD_CT_—connective tissue optical density; INT—intact group; RT(C)—repeated heat exposure control group; RT(X)—repeated heat exposure experimental group.

## Data Availability

The datasets presented in this article are not publicly available due to ongoing research. However, they can be made available by the authors upon reasonable request.

## Abbreviations

The following abbreviations are used in this manuscript:

MC	Mast cell
SSC	Spermatogonial stem cell
OD	Optical density
CT	Connective tissue
DI	Degranulation index
HMC-1	Human mast cell line
ATP	Adenosine three phosphate
TRPV1	Transient receptor potential vanilloid 1
TRPV2	Transient receptor potential vanilloid 2
H_2_R	Histamine receptor 2
PAR2	Protease-activated receptor 2

## Appendix A

### Appendix A.1

The total number of animals used in this study was confirmed using the resource equation method. According to this method, any sample size that yields an error degrees of freedom (DF) between 10 and 20 in the analysis of variance (ANOVA) is considered adequate. The error DF in one-way ANOVA is calculated using the following equation:

DF = Total number of animals − Total number of groups

Therefore, in the present study, which included four experimental groups, the minimum number of animals required was 14, and the maximum number was 24. In total, we used 31 animals, which exceeds the upper limit recommended by this method.

### Appendix A.3

**Table A1:** Post-hoc pairwise comparisons of mast cell populations among different reproductive organs presented in Table 1.

Parameter		Testis vs. Epididymis	Testis vs. Seminal Vesicle	Epididymis vs. Seminal Vesicle
MC density		0.065 (0.562)	0.0003 (1.073)	0.065 (0.507)
Synthetic activity		0.011 (0.700)	0.001 (0.957)	0.140 (0.253)
Degranulation index		0.768 (0.174)	0.768 (0.099)	0.768 (0.273)
Area		0.009 (0.720)	0.001 (0.941)	0.165 (0.217)
Perimeter		0.006 (0.759)	0.002 (0.908)	0.221 (0.145)
Maturation degree of MC granules	Immature	0.179 (0.55)	0.392 (0.30)	0.392 (0.257)
	Intermediate	0.340 (0.357)	0.340 (0.436)	0.842 (0.073)
	Mature	0.647 (0.206)	0.647 (0.158)	0.647 (0.363)

Data are presented as *q*-value (effect size *r*). Significant differences were determined through Dunn’s test and were adjusted using the two-stage step-up method of Benjamini, Krieger, and Yekutieli (*q* < 0.05). Effect size *r* was interpreted as follows: ~0.1 = small effect; ~0.3 = medium effect; ≥0.5 = large effect. MC—mast cells.

**Table A2:** Pairwise comparisons of morphofunctional parameters of mast cells among experimental groups presented in Table 2, Figure 3 and Table 3.

	Parameter		INT vs. ST	INT vs. RT(C)	INT vs. RT(X)	RT(C) vs. RT(X)
Testis	MC density		0.792 (0.13)	0.024 (0.68)	0.491 (0.21)	0.005 (0.89)
	Synthetic activity		0.195 (0.50)	0.578 (0.24)	0.488 (0.43)	0.578 (0.19)
	Degranulation index		0.009 (0.93)	0.099 (0.44)	0.002 (1.01)	0.077 (0.57)
	Area		0.082 (0.67)	0.288 (0.51)	0.497 (0.22)	0.497 (0.29)
	Perimeter		0.126 (0.60)	0.445 (0.32)	0.445 (0.35)	0.994 (0.02)
	Maturation degree of MC granules	Immature	0.643 (0.20)	0.216 (0.47)	0.216 (0.54)	0.873 (0.07)
		Intermediate	>0.999 (0.00)	0.334 (0.39)	0.200 (0.59)	0.571 (0.19)
		Mature	>0.999 (0.04)	0.688 (0.29)	0.688 (0.25)	0.932 (0.04)
Epididymis	MC density		0.841 (0.12)	0.724 (0.11)	0.005 (0.89)	0.007 (0.78)
	Synthetic activity		>0.999 (0.04)	0.991 (0.02)	0.282 (0.45)	0.282 (0.43)
	Degranulation index		0.057 (0.76)	0.277 (0.27)	0.012 (0.87)	0.059 (0.60)
	Area		0.548 (0.28)	0.164 (0.51)	0.150 (0.63)	0.760 (0.11)
	Perimeter		>0.999 (0.04)	0.320 (0.40)	0.320 (0.47)	0.874 (0.07)
	Maturation degree of MC granules	Immature	0.048 (0.71)	>0.999 (0.01)	>0.999 (0.03)	>0.999 (0.02)
		Intermediate	>0.999 (0.03)	0.480 (0.41)	0.579 (0.23)	0.579 (0.19)
		Mature	0.048 (0.71)	0.929 (0.10)	0.929 (0.04)	0.929 (0.06)
Seminal vesicle	MC density		0.329 (0.40)	0.559 (0.19)	0.375 (0.36)	0.265 (0.55)
	Synthetic activity		0.126 (0.60)	0.214 (0.15)	0.003 (0.91)	0.009 (0.76)
	Degranulation index		0.004 (1.00)	0.139 (0.45)	0.012 (0.83)	0.162 (0.38)
	Area		>0.999 (0.00)	0.006 (0.83)	0.296 (0.06)	0.007 (0.78)
	Perimeter		0.792 (0.13)	0.113 (0.63)	0.724 (0.12)	0.163 (0.52)
	Maturation degree of MC granules	Immature	0.421 (0.36)	0.059 (0.60)	0.367 (0.20)	0.023 (0.81)
		Intermediate	>0.999 (0.04)	0.283 (0.54)	0.320 (0.40)	0.705 (0.13)
		Mature	0.421 (0.36)	0.504 (0.29)	0.504 (0.22)	0.327 (0.51)

Data are presented as *p*-value (effect size *r_rb_*) for INT vs. ST; *q*-value (effect size *r*) for INT vs. RT(C), INT vs. RT(X), and RT(C) vs. RT(X). Statistical significance was determined using the Mann–Whitney U test for INT vs. ST (*p* < 0.05) and Dunn’s post-hoc test for INT vs. RT(C), INT vs. RT(X), and RT(C) vs. RT(X), with *p*-values adjusted using the two-stage step-up method of Benjamini, Krieger, and Yekutieli (*q* < 0.05). Effect sizes were interpreted as follows: ~0.1 = small effect; ~0.3 = medium effect; ≥0.5 = large effect. INT—intact group; ST—single heat exposure group; RT(C)—repeated heat exposure control group; RT(X)—repeated heat exposure experimental group; MC—mast cells.

**Table A3:** Spearman correlation analyses shown in Figure 6.

Correlation Matrix	Correlation Pair	Spearman *r*	95% CI	*p*-Value	*q*-Value
Testicular mast cells and epididymal sperm	MC density—Degranulation index	0.177	−0.301 to 0.584	0.456	0.239
	MC density—Sperm concentration	−0.331	−0.683 to 0.144	0.153	0.101
	MC density—Sperm motility	−0.391	−0.717 to 0.077	0.089	0.066
	MC density—Sperm agglutination	−0.185	−0.589 to 0.294	0.435	0.239
	Degranulation index—Sperm concentration	−0.511	−0.783 to −0.075	0.021	0.022
	Degranulation index—Sperm motility	−0.551	−0.804 to −0.129	0.012	0.021
	Degranulation index—Sperm agglutination	−0.450	−0.751 to 0.005	0.047	0.041
	Sperm concentration—Sperm motility	0.700	0.362 to 0.876	0.001	0.003
	Sperm concentration—Sperm agglutination	0.528	0.098 to 0.792	0.017	0.022
	Sperm motility—Sperm agglutination	0.559	0.141 to 0.808	0.010	0.021
Epididymal mast cells and ejaculated sperm	MC density—Degranulation index	0.689	0.258 to 0.892	0.006	0.012
	MC density—Sperm concentration	−0.454	−0.790 to 0.093	0.090	0.084
	MC density—Sperm motility	−0.508	−0.815 to 0.022	0.055	0.070
	MC density—Sperm agglutination	−0.263	−0.692 to 0.303	0.350	0.220
	Degranulation index—Sperm concentration	−0.733	−0.908 to −0.338	0.003	0.012
	Degranulation index—Sperm motility	−0.434	−0.781 to 0.117	0.107	0.084
	Degranulation index—Sperm agglutination	−0.322	−0.724 to 0.244	0.248	0.174
	Sperm concentration—Sperm motility	0.629	0.156 to 0.867	0.014	0.022
	Sperm concentration—Sperm agglutination	0.439	−0.111 to 0.783	0.105	0.084
	Sperm motility—Sperm agglutination	0.706	0.287 to 0.898	0.004	0.012
Seminal vesicle mast cells and ejaculated sperm	MC density—Degranulation index	−0.039	−0.552 to 0.495	0.893	0.656
	MC density—Sperm concentration	−0.248	−0.684 to 0.317	0.369	0.339
	MC density—Sperm motility	−0.494	−0.809 to 0.042	0.063	0.117
	MC density—Sperm agglutination	−0.633	−0.869 to −0.163	0.013	0.034
	Degranulation index—Sperm concentration	−0.375	−0.752 to 0.186	0.168	0.176
	Degranulation index—Sperm motility	−0.445	−0.786 to 0.103	0.098	0.129
	Degranulation index—Sperm agglutination	−0.049	−0.559 to 0.488	0.875	0.656
	Sperm concentration—Sperm motility	0.629	0.156 to 0.867	0.014	0.034
	Sperm concentration—Sperm agglutination	0.439	−0.111 to 0.783	0.105	0.129
	Sperm motility—Sperm agglutination	0.706	0.287 to 0.898	0.004	0.029
Testosterone with mast cell parameters in all reproductive organs and sperm parameters	Testosterone—Testicular MC density	−0.338	−0.687 to 0.136	0.144	0.272
	Testosterone—Testicular MC degranulation index	−0.660	−0.857 to −0.295	0.002	0.017
	Testosterone—Epididymal MC density	−0.262	−0.640 to 0.218	0.265	0.385
	Testosterone—Epididymal MC degranulation index	−0.403	−0.724 to 0.062	0.078	0.211
	Testosterone—Seminal vesicles MC number	−0.068	−0.506 to 0.398	0.777	0.771
	Testosterone—Seminal vesicles MC degranulation index	−0.406	−0.726 to 0.059	0.076	0.211
	Testosterone—Epididymal sperm concentration	0.340	−0.135 to 0.688	0.142	0.060
	Testosterone—Epididymal sperm motility	0.488	0.044 to 0.771	0.029	0.015
	Testosterone—Epididymal sperm agglutination	0.222	−0.258 to 0.614	0.348	0.122
	Testosterone—Ejaculated sperm concentration	0.527	0.004 to 0.824	0.046	0.105
	Testosterone—Ejaculated sperm motility	0.171	−0.389 to 0.638	0.540	0.510
	Testosterone—Ejaculated sperm agglutination	0.409	−0.147 to 0.769	0.135	0.142
	Testosterone—Lecithin granules	0.500	−0.034 to 0.812	0.061	0.105

*p*-values were adjusted using the two-stage stepwise method of Benjamini, Krieger, and Yekutieli and statistical significance was defined as *q* < 0.05. Correlation strength was interpreted as follows: weak |*r*| < 0.3; moderate: 0.3 ≤ |*r*| < 0.5; strong: |*r*| ≥ 0.5). MC—mast cells.
